# Natural SilibininLinoleate: A Protective Antioxidant in Edible Vegetable Oils

**DOI:** 10.3390/foods14193430

**Published:** 2025-10-06

**Authors:** Cristina Adriana Dehelean, Sergio Liga, Mariana-Atena Poiana, Ileana Cocan, Dorina Coricovac, Liliana Cseh, Mariana Suba, Ersilia Alexa

**Affiliations:** 1 Faculty of Pharmacy, “Victor Babes” University of Medicine and Pharmacy Timisoara, Eftimie Murgu Square, No. 2, 300041 Timisoara, Romania; cadehelean@umft.ro (C.A.D.); dorinacoricovac@umft.ro (D.C.); 2 Research Center for Pharmaco-Toxicological Evaluations, Faculty of Pharmacy, “Victor Babes” University of Medicine and Pharmacy Timisoara, Eftimie Murgu Square, No. 2, 300041 Timisoara, Romania; 3 Doctoral School, University of Life Sciences “King Mihai I” from Timisoara, Aradului Street No 119, 300645 Timisoara, Romania; marianapoiana@usvt.ro; 4 Faculty of Chemical Engineering, Biotechnologies and Environmental Protection, Politehnica University Timisoara, Vasile Pârvan No. 6, 300223 Timisoara, Romania; sergio.liga96@gmail.com; 5 Faculty of Food Engineering, University of Life Sciences “King Mihai I” from Timisoara, Aradului Street No 119, 300645 Timisoara, Romania; ileanacocan@usvt.ro; 6 “Food Science” Research Center, University of Life Sciences “King Mihai I” from Timisoara, Aradului Street No. 119, 300645 Timisoara, Romania; 7 Romanian Academy, “Coriolan Dragulescu” Institute of Chemistry, Mihai Viteazu No. 24, 300223 Timisoara, Romania; lcseh@acad-icht.tm.edu.ro (L.C.); marianasuba@gmail.com (M.S.)

**Keywords:** oxidative degradation, sunflower oil, fatty acid profile, FTIR, p-anisidine value, peroxide value

## Abstract

This study evaluated the potential of silibinin linoleate (SL), a natural derivative of silibinin, as an antioxidant to improve the thermal stability of sunflower oil (SF). SL was synthesized through green technology by enzymatic esterification, using mild reaction conditions. SL was added to high-oleic SF samples at three concentrations (200, 400, and 600 ppm), and the oils were subjected to heating at 180 °C for 4 and 8 h. Oxidative stability, fatty acid composition, and nutritional indices were analyzed. The results showed that 600 ppm SL provided the strongest antioxidant effect, significantly reducing oxidation parameters after 8 h of heating, in addition to the following values: peroxide value (PV) 14.22 ± 0.31 meq O_2_/kg, p-anisidine value (p-AV) 22.85 ± 0.34, inhibition of oxidation (IO) 56.41 ± 0.31%, and total oxidation value (TOTOX) 51.30 ± 0.39. FTIR spectroscopy confirmed that SL effectively protected the triglyceride structure and limited the formation of oxidation by-products. SL demonstrated a protective effect against thermal oxidation in sunflower oil, with its efficacy being clearly dose-dependent. At 600 ppm, SL showed comparable or superior activity to BHT. However, this effect was specific to the highest tested concentration and does not indicate superiority across all concentrations. These findings suggest that SL has potential as a natural antioxidant for improving oil stability, but further studies are needed to validate SL as a practical and scalable alternative to synthetic antioxidants in the food industry.

## 1. Introduction

Traditionally, the preservation of vegetable oils has relied on synthetic antioxidants such as BHT (butylated hydroxytoluene), BHA (butylated hydroxyanisole), and TBHQ (tert-butylhydroquinone), which prevent lipid oxidation by donating hydrogen atoms to peroxyl radicals [1]. However, in recent years, concerns about their potential toxicity and bioaccumulation have shifted attention toward natural alternatives with comparable or superior efficacy [2].

In the context of increasing interest in natural food preservation and replacing synthetic antioxidants with safe and effective alternatives, recent research has focused on natural bioactive molecules with high antioxidant properties. A notable example is silibinin, the main active constituent of *Silybum marianum* (milk thistle) is a flavonolignan with strong antioxidant, hepatoprotective, and anti-inflammatory properties [3].

Recent studies show that *Silybum marianum* seed oil contains a natural blend of PUFAs (ω-3, ω-6, and ω-9), along with antioxidant such as vitamin E and carotenoids, and exhibit oxidative stability exceeding 14 h, making it a promising a functional oil [4]. A study on silymarin, a standardized rich in silibinin, showed that at 100–450 ppm it effectively inhibited sunflower oil oxidation at level comparable to synthetic BHT, thereby extending fat shelf life [5]. In addition, silymarin exhibited antimicrobial activity against certain bacteria (e.g., *Staphylococcus aureus*, *E. coli*) and molds (*Aspergillus* spp.), supporting its potential as a natural food preservative [5].

SIL (silibinin linoleate), an ester of silibinin, the main active component of silymarin, is synthesized by reacting silibinin with unsaturated fatty acids such as oleic (omega-9) and linoleic (omega-6) acids [6]. These esters were developed to improve the solubility and bioavailability of silibinin while providing antioxidant properties tailored to lipid-rich environments. The linoleic acid moiety increases fat solubility, giving the molecule high affinity and assuring uniform distribution in vegetable oils or other fat-based products [7]. Thus, SL can act directly at the site of lipid oxidation, showing greater effectiveness than hydrophilic antioxidants in fatty systems. In addition, it exhibits strong antioxidant activity through its ability to scavenge free radicals [8].

Beyond radical scavenging, silibinin derivatives can chelate pro-oxidant metal ions and stabilize lipid intermediates, offering multifunctional antioxidant protection [9]. SL esters are biodegradable, nontoxic, and fit consumer demand for “clean label” food additives [10,11]. Unlike synthetic antioxidants such as BHA and BHT, which are strictly regulated in food applications—maximum 200 mg/kg in EU (Regulation No. 1333/2008) [12]—plant-derived compounds such as SL face fewer restrictions when used as natural ingredients [13]. The safety evaluation of SL is essential before its potential introduction as a natural antioxidant in edible oils. Recent investigations have provided preliminary in vitro and in ovo data regarding its cytotoxicity and irritation potential, offering insight into safe concentration ranges and potential risks at higher doses [6]. Our previous study [6] evaluated SL and related silibinin fatty acid conjugates using a combination of cell culture assays, HET-CAM irritation testing, and computational toxicity predictions. Cell viability assays performed on three different cell lines—cardiac (H9c2), hepatic (HepaRG), and keratinocyte (HaCaT)—revealed that low concentrations of SL (1–25 µM) were well tolerated, with either neutral or slightly stimulatory effects on cell growth. However, at higher concentrations (50–100 µM), SL exhibited a clear cytotoxic response, with cell viability decreasing significantly. At the highest tested level (100 µM), SL reduced cell viability by approximately 25–30%, accompanied by morphological changes such as cell rounding, detachment, and nuclear fragmentation, indicative of apoptotic or necrotic processes [6]. Overall, these results suggest that SL is safe at low-to-moderate concentrations, but cytotoxic effects become apparent at levels above 50 µM.

Previous studies indicate that high concentrations (>500 ppm) may paradoxically exhibit pro-oxidant behavior, potentially accelerating lipid oxidation rather than inhibiting it, especially under prolonged heating or in the presence of transition metals such as iron or copper [14,15]. This phenomenon has been reported for various natural polyphenols and suggests the importance of dose optimization.

One challenge in applying natural phenolic esters like SL in edible oils is their limited solubility and stability under industrial processing conditions. Recent technological advances have focused on delivery systems that improve dispersion, enhance antioxidant efficacy, and reduce the required dosage. Among these, nanoemulsion-based systems have emerged as particularly promising [16].

Electrostatic interaction-driven nanoemulsions, for example, utilize charged biopolymers or surfactants to create stable droplets that can encapsulate phenolic compounds, protecting them from thermal degradation and oxidation [16]. These systems not only improve bioavailability but also control the release of the antioxidant during storage and cooking, allowing for a lower total concentration to achieve the same or even greater protective effect. Other approaches include liposomal encapsulation, protein–polyphenol complexes, and microencapsulation using polysaccharide matrices, which have been successfully applied to antioxidants such as catechins and curcumin [17].

In recent years, research on natural antioxidants has advanced significantly, aiming to find safe and effective alternatives to synthetic antioxidants used to protect vegetable oils from oxidation [18,19]. Tocopherols, considered benchmark natural antioxidants, have shown beneficial effects on the oxidative stability of oils; however, their efficacy decreases considerably under high-temperature frying conditions, requiring higher doses or optimized combinations for optimal results [19,20]. Similarly, rosemary extracts, rich in carnosic acid, are approved for use in the food industry and provide good protection against oxidation during storage [19]. However, several recent studies have shown that during frying, their efficiency decreases, especially in oils with a high polyunsaturated fatty acid content, such as sunflower oil [15,16].

The catechins in green tea represent another group of natural antioxidants that have a protective effect on oils rich in polyunsaturated fatty acids; however, their thermal stability is limited [14]. Curcumin has also attracted increased interest, with recent studies reporting significant improvements in the oxidative stability of oils at concentrations of 500–1000 ppm [21].

Compared to these natural antioxidants, silibinin linoleate (SL) is a new compound with increased lipophilicity and potential for direct integration into the lipid phase, which gives it an advantage over free silibinin [6]. However, the literature from the last five years indicates that studies regarding the application of SL in edible oils at industrial temperatures are still limited, with most research focusing on eco-friendly synthesis and chemical characterization, rather than on detailed comparative evaluations with established antioxidants. Therefore, silibinin linoleate esters represent a promising natural antioxidant for stabilizing edible oils, with both nutritional (antioxidant, hepatoprotective) and technological (preservative, stabilizer) benefits. The application of SL in food systems remains limited, with challenges including optimal incorporation methods, dosage standardization, and a lack of comprehensive studies comparing its performance with widely used antioxidants. This study aims to address these gaps by evaluating the effectiveness of SL in sunflower oil under thermal processing conditions and comparing its performance to a conventional synthetic antioxidant, BHT, focusing on using SL as an antioxidant and preservative agent in the vegetable edible oil industry.

## 2. Materials and Methods

### 2.1. Obtaining of Silibinin Linoleate by Green-Synthesis

The obtaining of silibinin derivatives through green technologies was achieved according to our previous research [6]. SL was obtained by dissolving Silibinin (SIL), (Sigma Aldrich, Merck KgaA, Darmstadt, Germany), and linoleic acid (LA) (Sigma Aldrich, Merck KgaA Darmstadt, Germany) in ration SIL:LA = 1:3 in acetone. The initial reaction was performed for 10–12 h at 50 °C under magnetic stirring at 250 rpm using a digital magnetic stirrer with heating (IKA C-MAG HS 7, IKA-Werke GmbH & Co. KG, Staufen, Germany). Esterification was initiated by adding Novozyme 435 (10,000 U/g) (Novonesis, Kongens Lyngby, Denmark). The reaction mixture was then transferred to an orbital shaker (Heidolph Unimax 1010, Heidolph Instruments, Schwabach, Germany) with a 6 mm sample and maintained at 50 °C and 250 rpm for 120 h to complete the enzymatic esterification. To avoid evaporation of volatile components such as acetone and to ensure reproducibility of reaction conditions, the vessels were equipped with Teflon-lined screw caps and additional sealing using laboratory-grade parafilm. No measurable changes in reaction volume were observed at the end of the process, confirming effective prevention of evaporation losses. Purification of the SIL-L compound was performed by column chromatography, and the bioconjugates was concentrated using a Loborota 4000 Efficient Rotary Evaporator (Heidolph Instruments, Schwabach, Germany). Final purification was achieved by recrystallization from acetone and methanol.

### 2.2. Preparation of Oil Samples

Three samples of oil were prepared by adding different concentrations of silibinin oleate (SL) (200, 400, 600 ppm) to a high-oleic sunflower oil provided by Soloverde S.A., Romania (SF): SFSL1, SFSL2, SFSL3. Additionally, a control sample (C) consisting of sunflower oil and a sample of oil with the addition of BHT at a concentration of 200 ppm of BHT (maximum legal admissible dose) were prepared. The mixtures were then homogenized using an ultrasonic bath (Bandelin Sonorex, Berlin, Germany) at 40 kHz for 45 min to ensure complete dispersion of the antioxidant compounds into the oil. During sonication, the bath temperature was maintained below 35 °C by intermittent cooling with cold water to prevent thermal degradation of the antioxidants and to ensure uniform cavitation throughout the sample.

### 2.3. The Thermal Treatment Applied to Oils

To examine the effect of heating on SF supplemented with SL and BHT, mixture of oil samples prepared as is presented in Section 2.3, were subjected to thermal treatment in a conventional oven (Esmach, Esmach Ali Group S.r.l., Grisignano, Italy) 1200 W, 50 Hz to a temperature of 180 °C, similar to the conditions specific to the food industry, for 4 and 8 h of frying. After each heating period, the samples were removed from the furnace, cooled, and analyzed.

The abbreviation of the samples according to the composition and the thermal treatment applied is presented in Table 1.

### 2.4. Determination of Peroxide Value (PV) and the Inhibition of Oil Oxidation (IO)

The peroxide index (PV) is a measure of primary oxidation compounds in oil, being an important indicator of the degree of oxidation to which the product has been subjected. Its determination is performed by the iodometric method, according to the standards for oil analysis [22]. The result obtained reflects the degree of oxidation of the oil; the higher the PV, the more oxidized and less stable the oil is according to ISO 3960:2017 [22].

The peroxide index is expressed in milliequivalents of active oxygen per kilogram of oil (meq O_2_/kg) and is calculated according to Equation (1).

PV = (V × N) × 1000/m(1)

where V: volume of sodium thiosulfate used in titration (mL); N: normality of the thiosulfate solution; m: mass of the oil sample (g).

The inhibition of oil oxidation (IO) was calculated according to Formula (2) [23].

IO (%) = (1 − PV increase of the sample/PV increase of the control) × 100(2)

### 2.5. Determination of p-Anisidine Value (p-AV)

The determination of p-AV provides complementary information to the peroxide index (which detects primary oxidation), which is an important parameter for assessing the quality and stability of lipid products. p-Anisidine reacts with unsaturated aldehydes (especially 2-alkenals) in oil, forming chromophoric compounds that absorb radiation in the ultraviolet range, with a maximum absorption at 350 nm. The difference between the absorbance of the solution before and after the reaction with p-anisidine is used to calculate the p-AV value [24].

To analyze the p-AV activity the method described by Poiana et al. was performed [23]. The p-AV value was calculated according to Equation (3).

p-AV = 25 × 1.2 × (A2 − A1)/W(3)

where A1: absorbance of the oil solution before the reaction (compared to pure isooctane); A2: absorbance after the reaction (compared to the blank with p-anisidine); W: mass of the oil sample (g); and 1.2: standardized correction factor according to the AOCS method [24].

### 2.6. Determination of Total Oxidation Value (TOTOX)

To assess the total degree of oxidative degradation of an oil, it is important not only to determine the primary oxidation compounds (peroxides) and secondary compounds (aldehydes) but also to correlate them into a single integrative index. In this sense, the TOTOX value is used, which is a parameter that reflects the overall state of oxidation of oils and fats. This value is calculated mathematically, not directly determined through a chemical reaction. It results from the combination of the peroxide value (PV), which expresses the content of peroxides—the main compounds resulting in the early stages of oxidation—with the p-anisidine value (p-AV), which indicates the presence of aldehydes formed in the later stages of degradation [25]. TOTOX value was calculated using the formula shown in Equation (4).

TOTOX = 2 × PV + p-AV(4)

where PV: peroxide value (meq O_2_/kg); p-AV: p-anisidine value.

Low TOTOX values (<10) indicate a good state of preservation and fresh, stable oil. Increased values (>20–30) suggest an advanced degree of oxidation, with possible negative effects on the taste, smell, and nutritional quality of the oil.

### 2.7. Fourier-Transform Infrared Spectroscopy (FTIR)

Fourier-transform infrared spectroscopy (FTIR) was used to characterize the functional composition and structural changes in the analyzed oils [26]. For the FTIR analysis, the oil samples were used in liquid form, without any additional pre-treatment. A few drops of each sample were placed directly on the analysis crystal of the ATR (Attenuated Total Reflectance) accessory of the FTIR spectrometer Nicolet Is50 FT-IR (Thermo Fisher Scientific, Waltham, MA, USA). After applying the sample, spectra were collected in the range of 4000–600 cm^−1^, at a resolution of 4 cm^−1^, averaging 32 scans for each analysis to achieve an optimal signal-to-noise ratio. The background spectrum was recorded before each sample, using air or a clean surface.

### 2.8. Determination of Fatty Acid Profile

For the determination of the fatty acid profile, a method based on the transesterification of fatty acids in the oil was used, followed by the analysis of the resulting methyl esters (FAME) by gas chromatography (GC) [27]. The FAME preparation followed the AOCS Official Method Ce 1h-05 (2020), which ensures complete and efficient methylation of fatty acids under mild conditions to minimize thermal degradation or volatilization of sensitive PUFA species. Although highly oxidized derivatives, such as hydroxy- or epoxy-fatty acids, might be partially lost or undetectable by conventional GC-FAME due to their altered volatility, previous studies have shown this method to be reliable for comparative analysis of fresh and oxidized oils [18,20]. Future work will include GC-MS analysis to directly identify and quantify specific oxidation products that may not be detected by standard GC-FAME.

Chromatographic conditions: The analyses were performed using Agilent GC (Agilent Technologies, Santa Clara, CA, USA) on an Agilent CP-Sil 88 for FAME column. The temperature program used was as follows: initial temperature of 170 °C, maintained for 10–11 min; subsequently, the temperature was increased by 7.9 °C/min to 200 °C, held for 1.27 min, then continued to increase at the same rate to 210 °C, where it was held for another 16 min. The injector temperature was set at 250 °C, and the detector temperature was set at 300 °C. The carrier gas had a flow rate of 1.2 mL/min (equivalent to 26 cm/s), with a split ratio of 100:1. The hydrogen flow rate was set to 30 mL/min, and the air flow rate was set to 300 mL/min. The sample volume injected was 1 µL. Fatty acids were identified based on the NIST 05 spectrum library using the normalization method by relating the peak area corresponding to a given compound to the total area of all peaks. All analyses were performed in triplicate.

### 2.9. Calculation of Fatty Acids Indices

Saturated fatty acids (SFAs) were calculated as the sum of C14:0–C24:0 (Equation (5)), monounsaturated fatty acids (MUFAs) as the sum of C16:1–C24:1 (Equation (6)), and polyunsaturated fatty acids (PUFAs) as the sum of C18:2, C18:3, and C20:4 (Equation (7)). Unsaturated fatty acids (UFAs) were calculated as the sum of MUFAs and PUFAs [28].

SFA (%) = ∑ (C14:0 + C16:0 + C17:0 + C18:0 + C20:0 + C22:0 + C24:0)(5)

MUFA (%) = ∑ C16:1 + C17:1 + C18:1 + C20:1 C22:1 + C24:1)(6)

PUFA (%) = ∑ (C18:2 n-6 + C18:3 n-6 + C18:3 n-3 + C18:4 + C20:5 + C22:6)(7)

Based on the identified fatty acids, the atherogenic index (AI), thrombogenic index (TI), the hypocholesterolemic/hypercholesterolemic ratio (HH), and Omega-6/Omega-3 ratio (n6/n3) were calculated using the following equations:

The atherogenic index (AI), a functional indicator reflecting the proatherogenic potential of the lipid fraction, was calculated using the formula shown in Equation (8) [29].

AI = {C12:0 + (4 × C14:0) + C16:0}/{n-6 PUFA + n-3 PUFA + MUFA}(8)

The thrombogenic index (TI), reflecting the pro-thrombotic potential of the lipid fraction, was calculated using the formula shown in Equation (9) [29].

TI = {C14:0 + C16:0 + C18:0}/{(0.5 × MUFA)+(0.5 × n-6 PUFA)+(3 × n-3 PUFA) + (n-3 PUFA/n-6 PUFA)}(9)

The hypocholesterolemic/hypercholesterolemic ratio (HH), which evaluates the potential effect of fats on cholesterol metabolism, was calculated using the formula displayed in Equation (10) [30].

HH = (C18:1 + ΣPUFA)/(C12:0 + C14:0 + C16:0)(10)

The omega-6/omega-3 (n6/n3) ratio was calculated using Equation (11) [30] and reflects the balance between pro-inflammatory and anti-inflammatory fatty acids.

n6/n3 = ([C18:2 n-6] + [C18:3 n-6] + [C20:2 n-6])/([C18:3 n-3] + [C20:5 n-3])(11)

The polyunsaturated fatty acid/saturated fatty acid ratio (PUFA/SFA), a functional indicator of fat quality reflecting the balance between polyunsaturated and saturated fatty acids, was calculated using the formula presented in Equation (12) [30].

PUFA/SFA = ΣPUFA/ΣSFA(12)

### 2.10. Statistical Analysis

Values are reported as the mean of three independent analyses ± standard deviation (SD). Statistical analysis was performed using one-way ANOVA, followed by Tukey’s post hoc test and Levene’s test for homogeneity of variances, to evaluate differences related to heat exposure duration (0, 4, and 8 h) and antioxidant supplementation. Differences were considered statistically significant at *p* < 0.05.

## 3. Results and Discussion

### 3.1. The Influence of Silibinin Linoleate on Oxidation Parameters of Sunflower Oil

The values presented in Table 2 reflect the evolution of the peroxide indices (PVs), para-anisidine (p-AV), inhibition of oil oxidation (IO), and total oxidation value (TOTOX) in sunflower oil (SF), with or without the addition of silibinin linoleate (SL), before and after thermal exposure for 4 and 8 h. A synthetic control, BHT, used as a reference antioxidant, is also included.

At the initial moment (0 h), no significant differences (*p* > 0.05), for PVs, were recorded across all groups (approx. 1.74–1.81 meq O_2_/kg), indicating a similar level of primary oxidation before thermal treatment, both in control samples without the addition of synthetic/natural antioxidants and in samples with the addition of BHT and SL at different concentrations.

After 4 h of heating, the control oil (C) recorded a significant increase in PV (21.78 meq O_2_/kg), suggesting an accentuated lipid oxidation. BHT significantly reduced this value (13.73 meq O_2_/kg), confirming its effectiveness as a synthetic antioxidant. SL demonstrated a dose-dependent protective effect—from 18.18 meq O_2_/kg (SFSL1) to 9.72 meq O_2_/kg (SFSL3). Thus, at the maximum concentration (600 ppm), SL had superior efficiency even compared to BHT.

After 8 h of thermal treatment the trends persisted, with increased values for all samples, but again, SFSL3 showed the best antioxidant protection (14.22 meq O_2_/kg), followed by BHT (17.09 meq O_2_/kg) and SFSL2 (18.25 meq O_2_/kg), while the control reached a maximum of 30.45 meq O_2_/kg.

These results show that SL effectively reduces the formation of hydroperoxides, indicating its potential as a natural substitute for synthetic antioxidants.

p-Anisidine is an aromatic compound used as a reagent for evaluating the degree of secondary oxidation of oils and fats, more specifically for detecting aldehydes, especially those of the alkenal type, which appear after the decomposition of peroxides. These compounds contribute to the unpleasant taste and smell of rancid oils. Low p-AV values (<10) indicate reduced secondary oxidation and good oil quality. High p-AV values (>20–30) suggest the accumulation of aldehydes and significant degradation of the oil, often associated with unpleasant odor or rancidity.

Indicator of oxidation secondary products, p-AV showed the same increasing trends over time, with notable differences between treatments. As in the case of the evolution of pV, there are no statistically significant differences for the oil without thermal treatment, between the control sample and the ones treated either with synthetic antioxidant (BHT) or natural (SL).

At 4 h after heating at 180 °C, p-AV value increased to 30.47 for control, but was reduced to 18.09 for SFSL3 and 19.56 for BHT, suggesting a protective effect of SL comparable to or even superior to that of the synthetic one. At 8 h again, the control had the highest p-AV (41.07), while SL at 600 ppm (22.85) remarkably limited the formation of aldehyde compounds.

It is observed that after heating for 8 h, all oils show p-AV values above 20, which suggests advanced degradation of the oil. However, after heating for 4 h, the samples additive with 400 and 600 ppm SL show values below the threshold of 20, compared to those of BHT and even lower in the case of the SFL3 variant, indicating pronounced antioxidant activity. The results obtained demonstrate the remarkable antioxidant efficiency of silibinin linoleate in sunflower oil, comparable to or even superior to BHT, particularly at a concentration of 600 ppm. This effect can be attributed to the amphiphilic nature of SL, resulting from the esterification of silibinin with linoleic acid, which facilitates its integration into the lipid matrix and, implicitly, increases the antioxidant activity by effectively protecting unsaturated fatty acids from thermal oxidation.

The study on the peroxide value of eight vegetable oils indicated that sunflower oil was the most susceptible to oxidation with a PV of sunflower oil that increased significantly (27.35-fold) after 30 days of treatment at 600 C [31]. In oils rich in polyunsaturated fatty acids (such as sunflower, corn, or soybean oil), the use of natural compounds led to a significant decrease in peroxide (PV), p-anisidine (p-AV) and TOTOX values during storage and exposure to elevated temperatures. These effects have been attributed not only to their free radical scavenging capacity but also to possible interactions with minor compounds present in the oil, which may favor the regeneration of the active form of the antioxidant [32].

In the scientific literature, the increased efficacy of lipophilic derivatives of phenolic compounds is supported by studies showing that structural modification of natural antioxidant molecules (through esterification with fatty acids) significantly enhances their solubility in the lipid phase and provides them with increased affinity for the lipid–oxygen interface [2,33]. These data support the use of SL as a promising natural alternative to synthetic antioxidants in formulations of edible oils, contributing to the extension of shelf life and increasing the oxidative stability of lipid-rich food products.

The results clearly demonstrate that linoleic silibinin has an effective antioxidant capacity in sunflower oil, reducing both primary oxidation (by lowering PV) and secondary oxidation (by lowering p-AV) in a dose-dependent manner. The concentration of 600 ppm (SFSL3) proved to be the most effective and even surpassed the effect of BHT in most cases. The use of natural antioxidants is increasingly common to reduce the risks associated with the long-term consumption of synthetic antioxidants.

The changes in the inhibition of sunflower oil oxidation during heat exposure following supplementation with SL and BHT (Table 2) show that the inhibition effect, after 4 and 8 h compared to the control, decreases in the order SFSL3>BHT>SFSL2>SFSL1.

For the comprehensive assessment of the oxidative stability of sunflower oil (SF) subjected to thermal treatment, the TOTOX index was used as an integrative parameter (Table 2). It provides a global estimate of the degree of oxidation, combining both primary oxidation (through the peroxide value—PV) and secondary oxidation (through the para-anisidine value—p-AV), according to the formula TOTOX = 2PV + p-AV [34].

At the initial moment, the TOTOX values were similar across all samples (6.38–6.59) and no significant differences (*p* > 0.05) were recorded, indicating a minimal and comparable state of oxidation of the oil before thermal exposure. This uniformity suggests that the addition of SL does not influence oxidation in the absence of thermal stress, nor does it generate its own oxidation products.

After 4 h of heating, the differences become evident. The control (C) recorded a TOTOX value of 74.03, reflecting accelerated oxidation under the influence of temperature. The sample treated with BHT showed a significantly lower value (47.01), confirming its antioxidant efficiency. In the case of SL, a dose-dependent effect was observed—SFSL1 (200 ppm): 62.70, SFSL2 (400 ppm): 49.95, SFSL3 (600 ppm): 37.53. The reduction in the TOTOX value in the case of SFSL3, to below-BHT levels, highlights the superior efficiency of SL at higher concentrations in preventing both primary and secondary oxidation.

After 8 h of thermal treatment, the trends observed at 4 h are amplified. The reference oil reached a very high TOTOX level (101.98), indicating severe oxidative degradation. BHT reduced the value to 59.77, while SL again showed a dose-dependent efficiency—SFSL1: 83.27, SFSL2: 63.24, SFSL3: 51.30. This significant decrease at SFSL3 supports the hypothesis that SL at 600 ppm provides robust and persistent antioxidant protection, superior to synthetic BHT. It is presumed that the antioxidant activity of SL is due to both its polyphenolic structure and its esterification with linoleic acid, which increases its affinity for the lipid matrix [33].

The evolution of TOTOX values demonstrates the effectiveness of SL as a natural antioxidant in inhibiting the thermal oxidation of sunflower oil. At a concentration of 600 ppm, SL proved to be superior to BHT in all points of measurement post-thermal treatment. This behavior aligns with recent studies highlighting the potential of lipophilic derivatives of phenolic compounds in food applications by increasing oxidative stability and safety for consumers [2].

**Figure 1:**
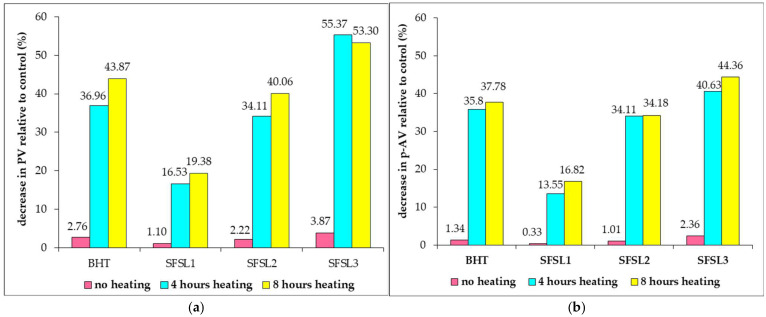
Decrease in oxidative activity of oils relative to the control in response to 4 and 8 h of heat exposure: (**a**) expressed as PV decrease (%); (**b**) expressed as p-AV decrease (%). Values calculated as difference between average values of samples and control, relative to the control value, are expressed in percentages.

Figure 1 presents the decrease in oxidative activity of oils related to the control expressed in % and calculated as difference between the values obtained for the samples with antioxidant and the control sample, reported to the control value, for each treatment separately.

Figure 1 shows a decrease in lipid oxidation compared to the control, both in the case of using synthetic additives, such as BHT, and silibilin linoleate, regardless of the added concentration. The rate of decrease in oxidative processes is lower in the case of oil samples not processed by heating and varies between 1.10 and 3.87% for PV, respectively, between 0.33 and 2.36% for p-AV. It should be noted that, at an additive concentration of 600 ppm, silibilin linoleate provides superior oxidation protection to BHT both in the unprocessed samples and in the samples subjected to heat treatment for 4 and 8 h, respectively. The percentage of inhibition of oxidative reactions is between 13.55 and 40.83% after 4 h of heat treatment for secondary oxidation reactions quantified by p-AV values, respectively, between 16.33 and 56.37% for primary oxidation processes expressed by PV. The increase in oxidative stability varies in the order: SFSL1<SFSL2<BHT<SFSL3 for both unprocessed samples and those heated at 180 °C, for 4 and 8 h. In the case of samples subjected to heat treatment for 8 h, the most pronounced effect of inhibiting the oxidative processes of the oil is noted, the decrease in PVs compared to the control being between 19.38 and 55.37%, respectively, of p-AV values between 16.82 and 44.36%, with maximum values in the case of sample SFSL3, confirming the antioxidant potential of silibilin linoleate on both primary and secondary oxidation reactions.

### 3.2. The Influence of Silibinin Linoleate (SL) on Oxidation Parameters of Sunflower Oil Based on FTIR Spectra

**Figure 2:**
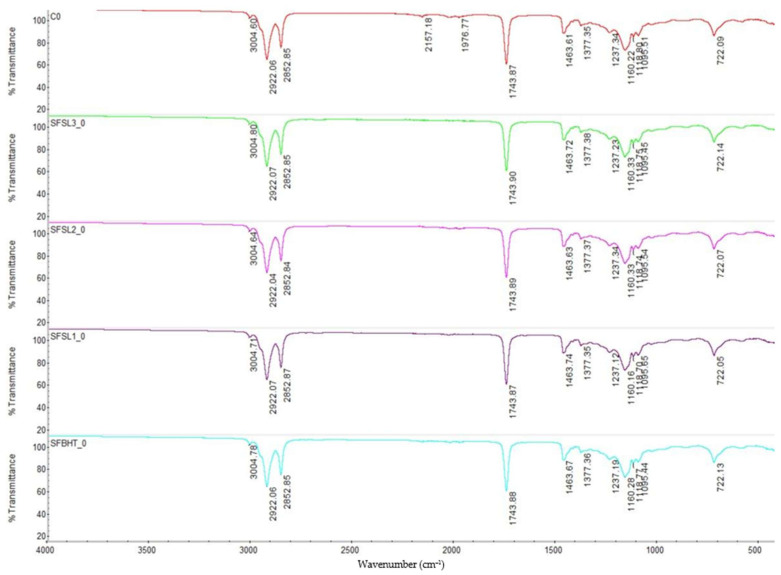
FTIR-spectra of samples of unprocessed oil samples: CO-control sample, sunflower oil (red line), SFBHT_0 sunflower oil additive with BHT (blue line), SFSL1_0 sunflower oil additive with 200 ppm silibilin linoleate (purple line), SFSL2_0 sunflower oil additive with 400 ppm silibilin linoleate (pink line) and SFSL3_0 sunflower oil additive with 600 ppm (green line). Spectral range of 4000–400 cm^−1^, 32 scans at 4 cm^−1^ resolution.

The FTIR spectra obtained for sunflower oil and its additive variants (BHT and silibinin linoleate) show common characteristics, specific to triglyceride-type lipid compounds (Figure 2). The main bands identified are 3004 cm^−1^ characteristic to the stretching vibrations =C–H specific to allylic and cis-diene groups present in unsaturated fatty acids (especially linoleic acid, the majority in sunflower oil), 3004–3007 cm^−1^ characteristic for C=C indicating unsaturated fatty acids, especially linoleic (predominant in sunflower oil, 2922 and 2853 cm^−1^ stretching vibrations of methylene groups (–H_2_–) characteristic of long aliphatic chains, 1743 cm^−1^ for stretching vibrations of carbonyl groups (C=O) in esters, characteristic of triglycerides, 1463 and 1377 cm^−1^ specific bands for deformation vibrations of methylene and methyl groups and 1237, 1160–1100 cm^−1^ for C–O stretching vibrations of the ester structure of triglycerides and 722 cm^−1^ band associated with long chains of –(CH_2_)n–, specific to the ordered aliphatic structure [18]. A specific broad –OH band is not clearly highlighted, which is expected, as there is no degradation or formation of oxygenated compounds (e.g., alcohols, hydroperoxides) yet. The triglyceride structure is intact. No significant signals for oxidants or oxidation products are observed.

The control sample (CO) shows the typical spectrum of sunflower oil, rich in unsaturated fatty acids. SFBHT0 (oil with BHT) does not show major differences at time 0, confirming that the synthetic antioxidant BHT does not induce structural changes detectable by FTIR in the initial lipid matrix. SFSL1_0, SFSL2_0, SFSL3_0 (oil with silibinin linoleate 200–600 ppm) show spectra similar to the control, maintaining the same characteristic bands of triglycerides. This suggests that the addition of silibinin linoleate does not modify the functional structure of lipid esters detectable by FTIR at the initial time. The intensity and position of the bands remain virtually unchanged between samples, confirming that neither BHT nor silibinin linoleate react initially with the lipid matrix but only act as oxidation inhibitors under subsequent processing or storage conditions [34].

At time 0 (unprocessed thermally), all samples exhibit FTIR spectra characteristic of triglycerides from sunflower oil, without major structural changes caused by the addition of BHT or linoleate silibinin. FTIR spectroscopy confirms that the introduced antioxidants do not alter the basic chemical structure of the oil but integrate into the matrix as protective agents against oxidation, with their effects to be highlighted under conditions of oxidative stress (heating, prolonged storage) [1].

**Figure 3:**
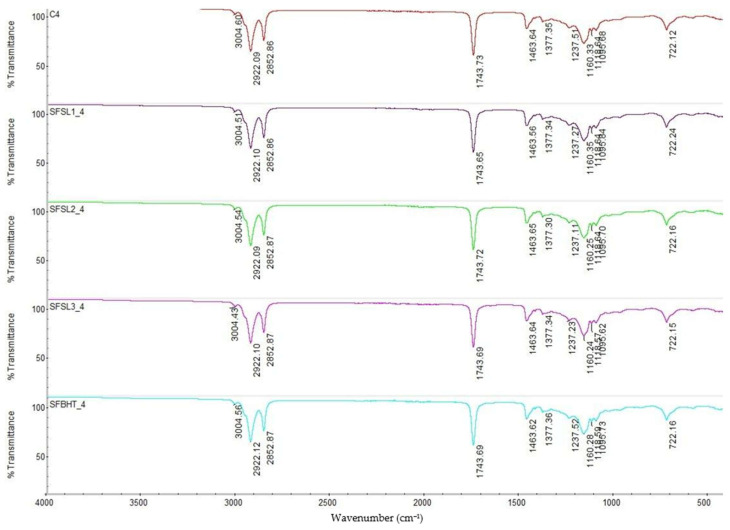
FTIR spectra of samples heat-processed at 180 °C for 4 h: C0-control sample, sunflower oil (red line), SFBHT4 sunflower oil additive with BHT (blue line), SFSL1_4 sunflower oil additive with 200 ppm silibilin linoleate (purple line), SFSL2_4 sunflower oil additive with 400 ppm silibilin linoleate (green line), and SFSL3_4 sunflower oil additive with 600 ppm (pink line). Spectral range of 4000–400 cm^−1^, 32 scans at 4 cm^−1^ resolution.

After 4 h of heating at 180 °C (Figure 3), the spectra show the same absorption regions characteristic of triglycerides but with some subtle changes compared to the spectra at time 0 (unheated). The bands at 3004 cm^−1^ (=C–H), characteristic for unsaturated fatty acids and 2922/2853 cm^−1^ (–CH_2_–) are still evident, but slight decreases in intensity appears, which suggests a decrease in the content of unsaturated fatty acids due to thermal oxidation and isomerization [35]. The band characteristic for C=C (~3004–3007 cm^−1^) is maintained, but with reduced intensity confirming the partial oxidation of unsaturated fatty acids. Regarding the –OH group (3000–3600 cm^−1^): a slight broadening of the band around 3000–3400 cm^−1^ appears, indicating the initial formation of hydroperoxides and fatty alcohols, more noticeable in the C4 sample. The beginning of thermal oxidation is observed in this stage. Samples with antioxidants (BHT and silibinin linoleate, especially at 400 ppm) better preserve the esters and C=C bonds. For the control sample C4, the intensity of bands 1743.65–1743.73 cm^−1^ is relatively stable, but slight deviations occur, which indicates structural changes through the oxidation and formation of secondary compounds (aldehydes, ketones, oxidized fatty acids). The presence of antioxidants (BHT and silibinin linoleate) contributes to maintaining the stability of the ester group, preventing massive degradation of triglycerides [36].

The methylene and methyl deformation bands (region 1465–1377) do not show major changes, but their slightly modified ratio may suggest fragmentation of aliphatic chains by oxidation. Region 1237–1100 cm^−1^ (C–O ester, stretching vibrations) is sensitive to lipid oxidation. In the control sample C4, intensifications and slight shifts in the bands are observed, a sign of the formation of oxygenated compounds. The samples with BHT and silibinin linoleate (especially SFSL3_4) present a profile closer to the initial one, which confirms the efficiency of antioxidants in protecting the triglyceride structure [1]. This band, associated with the elongation of hydrocarbon chains, is maintained in all samples. Its persistence indicates that the aliphatic chains are not completely degraded, even after 4 h of heat treatment.

Compared all the sample after 4 h of thermal process, it can be observed that SFSL3_4 (600 ppm silibinin linoleate) shows the highest structural stability, with bands almost identical to the sample at time 0. This confirms the superior efficiency of silibinin linoleate at high concentrations compared to BHT.

FTIR spectroscopy demonstrates that heating sunflower oil at 180 °C for 4 h causes structural changes associated with lipid oxidation, especially at the level of unsaturated fatty acid and ester groups. The addition of antioxidants (BHT and silibinin linoleate) limits these transformations, with a concentration-dependent protective effect. Silibinin linoleate at 600 ppm proves more effective than BHT in maintaining the structural integrity of triglycerides.

**Figure 4:**
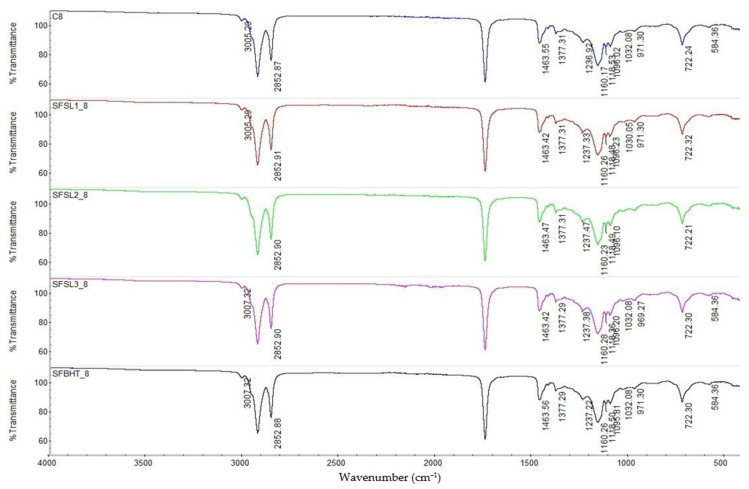
FTIR spectra of samples heat-processed at 180 °C for 8 h: C0-control sample, sunflower oil (blue line), SFBHT8 sunflower oil additive with BHT (dark blue line), SFSL1_8 sunflower oil additive with 200 ppm silibilin linoleate (red line), SFSL2_8 sunflower oil additive with 400 ppm silibilin linoleate (green line) and SFSL3_8 sunflower oil additive with 600 ppm (pink line). Spectral range of 4000–400 cm^−1^, 32 scans at 4 cm^−1^ resolution.

After 8 h of heating at 180 °C (Figure 4), the bands at 3007–3005 cm^−1^ (stretching =C–H of unsaturated fatty acids) and 2922/2852 cm^−1^ (stretching –CH_2_–) are visible, but compared to the spectra at time 0 and 4 h, their intensity decreases. This indicates advanced degradation of unsaturated fatty acids by thermal oxidation and formation of free radicals [36].

Regarding C=C (~3007–3005 cm^−1^) significantly reduced intensity in all samples, especially in SFSL1_8 was observed as a sign of the oxidation of unsaturated fatty acids. For –OH (3000–3600 cm^−1^) evident presence of a broad band, especially in C8 was observed, indicating the accumulation of secondary oxygenated products: hydroperoxides, alcohols, carboxylic acids. In this stage, oxidation is advanced. The disappearance of the C=C signal and the broadening of the –OH band confirm the transformation of PUFAs into oxidized compounds.

At 8 h, the band characteristic of the carbonyl group (C=O, triglyceride esters ~1740 cm^−1^) shows a decrease in intensity and slight shifts in all samples, more evident in the C8 control a sign of the accumulation of aldehydes and oxidized fatty acids. This phenomenon is an indicator of the hydrolysis and oxidation of triglycerides, leading to the formation of aldehydes, ketones, and oxygenated fatty acids [31]. In samples with antioxidants (especially SFSL3_8), the band is better preserved, which confirms the protective role of phenolic compounds and BHT. The results highlighted that the carbonyl band is a sensitive marker of thermal degradation; the addition of silibinin linoleate at 600 ppm maintains the triglyceride structure better than BHT.

The bands at 1463 cm^−1^ and 1377 cm^−1^ are present in all samples, but their intensity is lower at C8, suggesting fragmentation of aliphatic chains by oxidation reactions.

The control sample C8 shows significant changes in region 1237–1100 cm^−1^, especially higher intensity and slight shifts, a sign of the accumulation of secondary oxygenated compounds (epoxides, peroxides, fatty alcohols). In the samples with BHT and silibinin linoleate, these changes are attenuated, and the spectra are closer to the initial form. Sample SFSL3_8 (600 ppm) is the most stable, confirming that the protective effect of silibinin linoleate is concentration-dependent. The band at 722 cm^−1^, associated with the ordered structures of long hydrocarbon chains, persists in all samples, but with a decrease in intensity at C8. In samples with antioxidants, the band remains stronger, suggesting partial protection of the triglyceride structure against advanced oxidation.

The appearance of additional bands in the 970–980 cm^−1^ and 584 cm^−1^ area (visible at C8 and oxidized samples) is associated with the formation of secondary oxidation products and lipid polymers, a sign of advanced thermal oxidation [31].

The experimental results highlight that C8 (heat-processed control) show the most obvious signs of degradation, namely decrease in C–H and C=O bands, intensification of the C–O region and appearance of secondary oxidation bands. The synthetic antioxidant SFBHT_8 (BHT) offers partial protection; the triglyceride structure is better preserved than in C8, but signs of oxidation are visible. SFSL1_8 and SFSL2_8 maintain moderate stability of the carbonyl and C–O groups, but the protective effect increases with concentration. The best structural stability profile, after 8 h of heating is due to SFSL3_8 (600 ppm), with a spectrum much closer to the initial one, which confirms the superior efficiency of silibinin linoleate compared to BHT under severe heat stress conditions.

### 3.3. Fatty Acid Composition of Oil Samples

Lipid oxidation plays a critical role in determining food quality during both processing and storage. This process, particularly in polyunsaturated fatty acids, leads to the development of undesirable flavors (rancidity), a decline in nutritional value, and a shortened shelf life of food products [35]. In parallel, the oxidative degradation of lipids and proteins can adversely affect the cooking properties, sensory attributes, and nutritional profile of foods such as rice, diminishing their overall market value [37].

The accumulation of oxidation products—whether primary, secondary, or tertiary—can reach levels that may pose risks to consumer health. This reaction is essentially influenced by the chemical structure of fatty acids, particularly by their degree of unsaturation.

Polyunsaturated fatty acids (PUFAs), such as linoleic acid and alpha-linolenic acid, are especially susceptible to oxidation due to the presence of multiple double bonds, which constitute vulnerable points for free radical attack [38]. In this context, antioxidants play an essential role in preventing or delaying lipid oxidation. They act through various mechanisms, such as trapping free radicals, interrupting chain reactions, or chelating pro-oxidant metal ions [39].

The major saturated fatty acids (SFAs) are palmitic acid (C16:0) and stearic acid (C18:0), which show a tendency to increase slightly over time, especially in the control at 8 h (e.g., C16:0: CO_0 = 3.982%, CO_8 = 4.476%; C18:0: CO_0 = 2.540%, CO_8 = 2.896%) (Table 3). The relative increase in SFAs reflects the degradation of unsaturated fatty acids and their conversion through oxidation and chain scission reactions, a phenomenon documented in recent research [40,41]. Antioxidants do not completely prevent the increase in SFA, but some samples (e.g., SFSL3_8, SFBHT_8) show values close to the initial ones, suggesting a partial protection against massive oxidation of UFAs.

Oleic (C18:1), palmitoleic (C16:1), eicosenoic (C20:1), and erucic (C22:1) acids represent the main monounsaturated acids (MUFAs). Oleic acid decreases significantly upon prolonged heating in control samples (CO_0 = 81.084% to CO_8 = 80.924%) and much more steeply in some samples with low antioxidant concentrations (e.g., SFSL1_8: 57.815%) (Table 3). Samples SFBHT_8 and SFSL3_8 maintain high oleic acid values (74.743% and 76.165%), confirming their protective role. The decrease in MUFAs is less pronounced than that of polyunsaturated fatty acids (PUFAs), confirming the greater stability of acids with a single double bond to thermal oxidation [35].

The main PUFAs detected were linoleic acid (C18:2) and α-linolenic acid (ALA, C18:3α), which are the most sensitive to oxidation. In the control, linoleic acid decreases from 6.004% (CO_0) to 3.502% (CO_8), and ALA from 0.127% to 0.044% (Table 3). Samples with antioxidants show slower rates of decrease, especially SFBHT_8 and SFSL3_8, which retain more PUFAs compared to SFSL1_8 or SFSL2_8. This behavior confirms that PUFA protection is a better indicator of antioxidant efficiency than the total percentage of UFAs.

Based on the obtained results, we can say that heating causes degradation of PUFAs and, to a lesser extent, MUFAs, with a relative increase in SFAs.

The antioxidants BHT (SFBHT) and the high-concentration natural extract (SFSL3) more effectively protect PUFAs and maintain higher oleic acid values, even though the total percentage of UFAs may be lower than in the control. Samples with low antioxidant concentrations (SFSL1) significantly lose PUFAs and MUFAs, confirming that the efficiency depends on both the type and dose of antioxidant.

**Figure 5:**
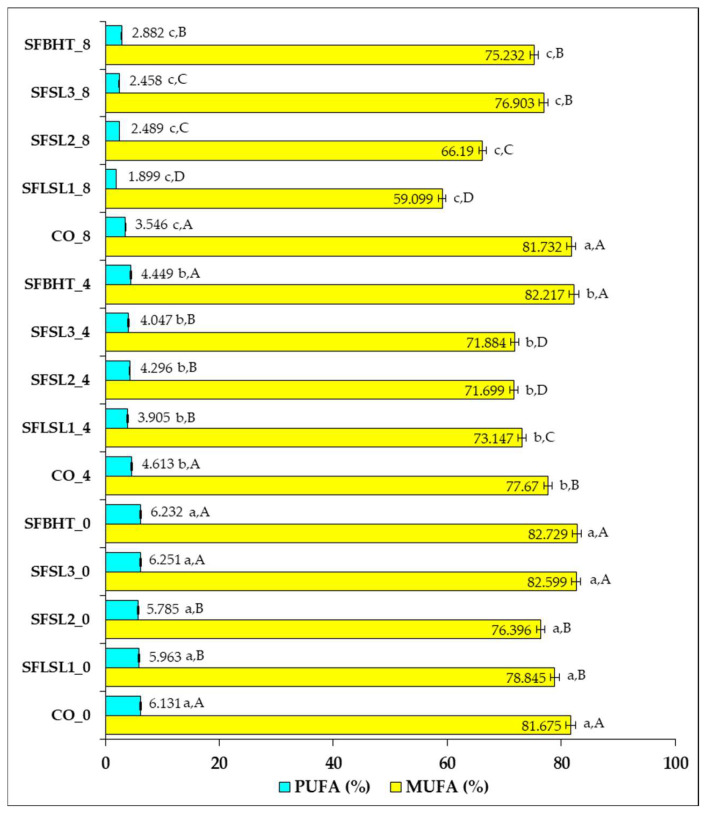
PUFA and MUFA content of oil samples (control—CO; antioxidant-supplemented—SFSL1, SFSL2, SFSL3, SFBHT) in response to heat exposure. Values represent the mean of three independent analyses ± standard deviation (SD). Bars with different letters are statistically different according to one-way ANOVA (*p* < 0.05). Lowercase letters indicate differences among samples with the same antioxidant dose across different thermal treatment durations (0, 4, and 8 h), whereas uppercase letters indicate differences among antioxidant-supplemented samples within the same exposure period.

Figure 5 illustrates the percentage changes in the composition of monounsaturated fatty acids (MUFAs) and polyunsaturated fatty acids (PUFAs) in sunflower oil subjected to heat treatments, with or without the addition of natural or synthetic antioxidants, at different time intervals (0, 4, and 8 h).

In the control sample (CO_0), the unheated oil shows a high content of MUFAs (81.675%) and a relatively low percentage of PUFAs (6.131%). The initial values are comparable to those of the samples with antioxidants added at time zero (e.g., SFSL1_0—78.845% MUFAs; 5.963% PUFAs), suggesting that the addition of antioxidants does not significantly modify the immediate composition of fatty acids.

After 4 h of heating, a slight decrease in MUFAs is observed in the control sample (CO_4—77.670%), concomitantly with a reduction in PUFAs (4.613%), which is in agreement with the literature, indicating thermal degradation of unsaturated fatty acids, especially PUFAs, due to their susceptibility to oxidation [38,42].

After 8 h of heating, the decreases become more pronounced. In CO_8, MUFAs reach 81.732%, but PUFAs decrease to 3.546%, indicating that polyunsaturated fatty acids were preferentially degraded during thermal oxidation, a phenomenon that has been widely documented in studies on the thermal stability of vegetable oils [43,44]. MUFAs, being more chemically stable, underwent fewer changes, and their relative percentage may even increase as a result of PUFA degradation.

Samples with added antioxidants show better PUFA values, for example, SFSL3_8 (76.903% MUFAs; 2.458% PUFAs) compared to SFSL1_8 (59.099% MUFAs; 1.899% PUFAs), suggesting a difference in the effectiveness of antioxidants depending on the type and concentration. This effect indicates that antioxidants limit the radical initiation and propagation reactions of lipid oxidation, especially preserving PUFAs, the fraction with high nutritional value [35].

Overall, the data confirm that heating the oil leads to a decrease in PUFAs to a greater extent than MUFAs, and the addition of antioxidants can slow down this process, but the effectiveness depends on the concentration and chemical structure. The results are consistent with the reports of Szabo et al. (2022) [44], who showed that PUFAs, having more double bonds, are much more sensitive to thermal oxidation than MUFAs.

**Figure 6:**
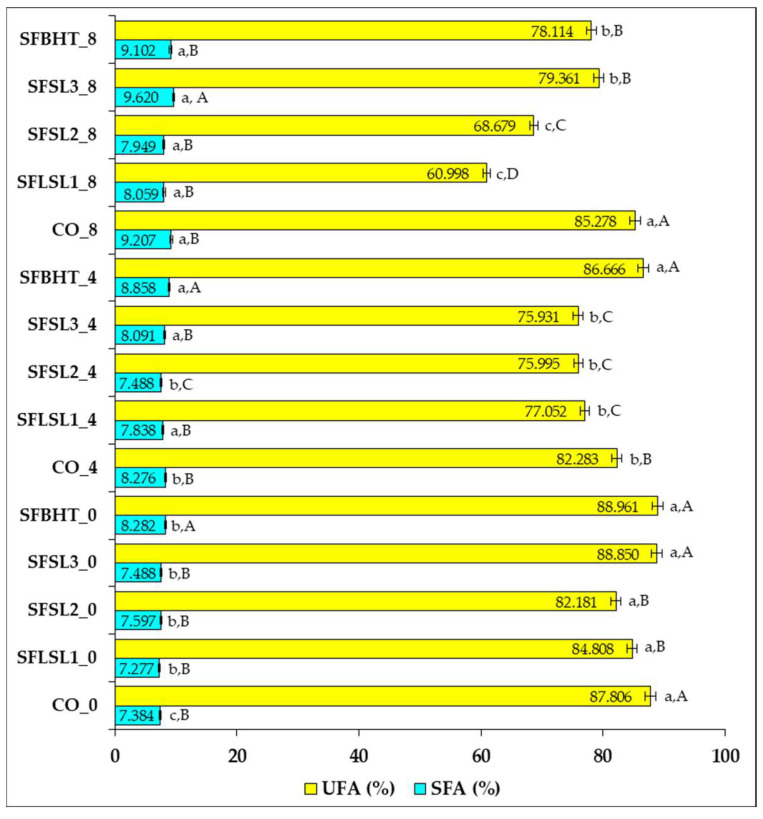
UFA and SFA content of oil samples (control—CO; antioxidant-supplemented—SFSL1, SFSL2, SFSL3, SFBHT) in response to heat exposure. Values represent the mean of three independent analyses ± standard deviation (SD). Bars with different letters are statistically different according to one-way ANOVA (*p* < 0.05). Lowercase letters indicate differences among samples with the same antioxidant dose across different thermal treatment durations (0, 4, and 8 h), whereas uppercase letters indicate differences among antioxidant-supplemented samples within the same exposure period.

Regarding SFA and UFA evolution after heating (Figure 6), the control samples (CO_0, CO_4, CO_8) show that unheated sunflower oil (CO_0) contains about 87.8% UFAs and 7.38% SFAs. After 4 h of heating (CO_4), UFAs slightly decrease to 82.28%, accompanied by a relative increase in SFAs (8.27%). After 8 h (CO_8), UFAs decrease to 85.28%, but SFAs increase to 9.21%. These changes reflect the tendency of unsaturated fatty acids to undergo thermal oxidation, which leads to the formation of more saturated compounds and the loss of double bonds [35,40].

Samples with synthetic or natural antioxidants (e.g., SFSL1, SFSL2, SFSL3, SFBHT) show variable UFA values after heating, depending on the type and concentration of the antioxidant. In general, at time zero, all samples have UFA levels comparable to the control (82–89%), confirming that the addition of antioxidants does not significantly alter the initial fatty acid composition.

After 4 h, moderate decreases in UFAs (75.93–86.67%) and corresponding increases in SFAs (7.49–8.86%) are observed in the samples with antioxidants, suggesting that antioxidants delay, but do not completely prevent, oxidation.

After 8 h, the differences become more pronounced; samples SFSL2_8 and SFSL1_8 show marked decreases in UFAs (68.68% and 60.99%, respectively), signaling reduced antioxidant efficacy under these conditions. Samples SFBHT_8 and SFSL3_8 retain higher levels of UFAs (78.11% and 79.36%), suggesting better protection against oxidation.

SFBHT (butylated hydroxytoluene) and SFSL3 (natural extract, higher concentration) demonstrated the best capacity to protect PUFAs and limit SFA growth after 8 h of heating. Even though the total percentage of UFAs is slightly lower than in the control, these samples preserved the original fatty acid structure to a greater extent and reduced the accumulation of oxidized compounds. This qualitative difference is essential from a nutritional and technological point of view, confirming recent conclusions on the importance of protecting PUFAs from oxidation, not just maintaining a raw percentage of UFAs [40,43].

**Figure 7:**
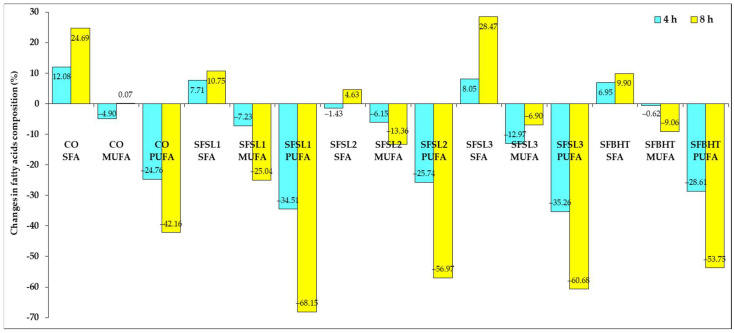
Change in the main classes of fatty acids—saturated (SFAs), monounsaturated (MUFAs), and polyunsaturated (PUFAs)—in oil samples (control—CO; antioxidant-supplemented—SFSL1, SFSL2, SFSL3, SFBHT) following 4 and 8 h of heat exposure. Values are calculated as the difference between average values of samples at 4 and 8 h and the average values of the same samples at initial time, relative to the value of the sample at initial time, expressed in percentages.

Figure 7 presents highlights the percentage changes in the main classes of fatty acids—saturated fatty acids (SFAs), monounsaturated fatty acids (MUFAs) and polyunsaturated fatty acids (PUFAs)—in sunflower oil, after 4 and 8 h of heating, for the control (CO) and samples treated with antioxidants (SFSL1, SFSL2, SFSL3, SFBHT).

In all samples, heating causes an increase in the SFA fraction, a change that is more evident after 8 h. This increase is explained by the superior thermal stability of SFAs and the accelerated degradation of unsaturated fatty acids, which leads to a relative increase in their proportion. MUFAs present mixed variations, with modest percentage changes, and the increases observed in some samples are attributable to the partial conversion of PUFAs into MUFAs through partial oxidation reactions.

PUFAs, the most susceptible to oxidation, registered the largest percentage decrease in all samples, a phenomenon accentuated at 8 h. The control (CO) shows a decrease in PUFAs of −42.16% after 8 h, a lower value than in most samples with antioxidants. In contrast, SFSL1 (−68.15%), SFSL2 (−56.97%) and SFSL3 (−60.68%) show much higher percentage losses, and SFBHT (−53.75%) has an intermediate degradation between the control and the other samples with antioxidants.

This discrepancy is explained by the fact that GC-FAME analysis quantifies only the remaining intact PUFAs, whereas oxidized fatty acids are converted into volatile fragments, aldehydes, and polymeric products that are no longer detected as PUFA peaks [18,20]. Consequently, the control oil appears to retain more PUFAs, but in reality, a significant fraction has already been oxidized to non-detectable forms. Meanwhile, antioxidant-treated oils show lower PV and p-AV, indicating slower oxidation, even though the formation of certain conjugated PUFA derivatives may result in slightly lower detectable PUFA levels.

**Figure 8:**
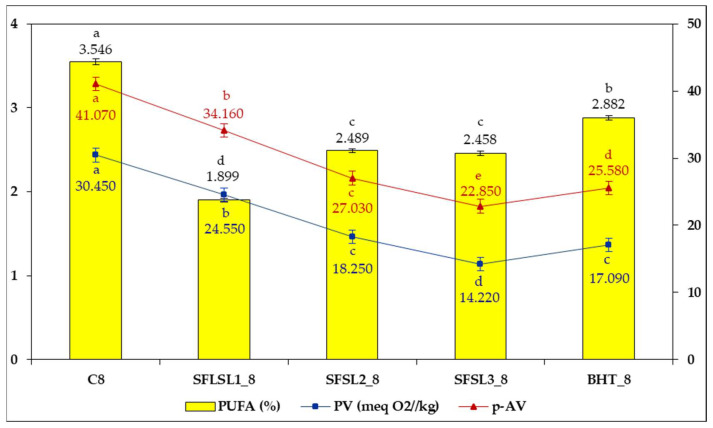
Integrated interpretation of the effect of antioxidants on fatty acid composition and oxidative stability (PV and p-AV) of oil samples (control—CO; antioxidant-supplemented—SFSL1, SFSL2, SFSL3, SFBHT) after 8 h of heat exposure. Bars and lines with different letters are statistically different according to one-way ANOVA (*p* < 0.05).

The correlation of GC analysis of fatty acids with primary (PV) and secondary oxidation (p-AV) data is presented in Figure 8.

After 8 h, CO shows extremely high values for both PV (30.45 meq O_2_/kg oil) and p-AV (41.07), indicating the significant accumulation of volatile peroxides and aldehydes—products characteristic of advanced PUFA oxidation. This means that a significant portion of the PUFAs apparently “preserved” in the chromatographic analysis are in fact partially or completely oxidized, with reduced nutritional value and potential pro-oxidative effect. In contrast, the samples with antioxidants, especially SFSL3 and BHT, although presenting a lower absolute amount of PUFAs than the control, have much lower PV and p-AV values. SFSL3 records the lowest PVs (14.22) and the lowest p-AV (22.85) of all the samples, closely followed by BHT (PV = 17.09; p-AV = 25.58). In samples with SL or BHT, antioxidants slow the rate of oxidation, preventing uncontrolled chain reactions and secondary product formation. They may also promote alternative reaction routes, such as the formation of stable conjugated isomers, which are measured differently or even counted as degraded PUFAs in GC. Thus, treated samples can show a greater apparent decrease in PUFAs but lower PVs and p-AV, indicating better preservation of oil quality. In the control, the absence of antioxidants allows for rapid oxidation with extensive hydroperoxide and aldehyde accumulation, leading to high PVs and p-AV values. These oxidation products accumulate faster than the measurable loss of PUFAs, explaining the discrepancy between the two metrics. These results indicate significant protection against both primary and secondary oxidation, implying the preservation of the chemical and functional integrity of the remaining PUFAs.

The samples SFSL1 and SFSL2 show intermediate performances, reducing PV and p-AV values compared to the control, but without reaching the efficiency observed with SFSL3 and BHT. The superior efficacy of SFSL3 can be attributed to both the higher concentration of bioactive compounds with antioxidant potential and their synergy with the lipid matrix, which limits the propagation of radical oxidation reactions.

Recent studies confirm that the fatty acid profile alone is not sufficient to assess oxidative degradation, as it only measures the remaining fatty acids, not the oxidation products [44]. Oxidation indices (PV, TOTOX) increase rapidly even when PUFAs decrease only moderately [18]. In control samples, uncontrolled oxidation produces toxic aldehydes and polymers, even if some of the PUFAs appear residual in GC [20,44].

FTIR analysis further confirms the data correlated with GC analysis and oxidation indices, as follows:

1. Detection of direct oxidation by the formation of carbonyl groups

During the oxidation of PUFAs, they form hydroperoxides and then aldehydes, ketones, and carboxylic acids. These transformations are visible in the FTIR by the appearance or increase in the intensity of the bands in the carbonyl region (~1740–1710 cm^−1^). The control (without antioxidants) shows intense bands at 1743–1745 cm^−1^, indicating the accumulation of hydroperoxides and aldehydes. This demonstrates advanced oxidation, even though the apparent level of residual PUFAs is higher by GC. In samples with antioxidants, this band is reduced, showing that oxidation was limited, even though residual PUFAs appear lower by GC.

2. Changes in the bands characteristic of conjugated C=C bonds in PUFAs

PUFAs have characteristic C=C (cis) bands at ~3010 cm^−1^ (stretching =CH). During oxidation, the intensity of the band decreases as the double bonds are consumed. New bands may appear at 990–970 cm^−1^, corresponding to trans isomers or conjugated systems formed during the oxidation process. This transition may explain why in GC we observe an apparently high residual level of PUFAs in the control. The oxidized double bonds no longer appear as PUFAs in GC but are indirectly detectable by the new FTIR bands. In samples with antioxidants, the consumption of PUFAs is slower, and the changes at 3010 cm^−1^ are less pronounced.

3. Detection of polymerization and formation of large mass compounds

In advanced oxidized oils, new bands appear in the 1000–1100 cm^−1^ region and increase in intensity in the 2850–2925 cm^−1^ region (C–H stretching of polymerized saturated chains). These changes indicate formation of polymers and oligomers, which are not detectable by GC as PUFAs or as volatile compounds and explains why the control may appear “rich” in PUFAs in GC, but FTIR shows massive oxidation through these new structures.

Thus, the integrated interpretation of the data shows that the evaluation of antioxidant efficacy should not be performed exclusively based on the percentage profile of fatty acids but by combining it with specific indicators of lipid oxidation and FTIR spectra. From this perspective, SFSL3 and BHT demonstrate a superior antioxidant efficacy to the control, by significantly reducing oxidative degradation and preserving the nutritional value of PUFAs.

The antioxidant effectiveness of silibinin linoleate (SL) can be directly linked to its specific molecular structure and the modifications introduced by esterification. Silibinin, the parent compound of SL, is a polyphenolic flavonolignan characterized by multiple phenolic hydroxyl groups, most notably a catechol moiety on ring B and a hydroxyl group at position 3 that is conjugated with a double bond and a carbonyl group. These structural elements are critical for hydrogen atom donation and stabilization of the resulting phenoxyl radical through resonance, enabling efficient peroxyl radical scavenging and interruption of lipid oxidation chains [45]. Esterification of silibinin with linoleic acid produces a derivative with enhanced lipophilicity, increasing its solubility and retention in the lipid phase of edible oils. This structural modification allows SL to localize preferentially at oil–air and oil–water interfaces, where lipid autoxidation is most active. Such localization ensures that SL is positioned precisely at the initiation sites of lipid peroxidation, thereby increasing the likelihood of intercepting lipid radicals. This mechanism reflects recent findings that the partitioning behavior and interfacial positioning of phenolic antioxidants are key determinants of their performance in bulk oils and frying systems, complementing and refining the classic polar paradox theory [45,46]. In practical terms, SL acts as a chain-breaking antioxidant, neutralizing peroxyl radicals (ROO•) and converting them into more stable lipid hydroperoxides (ROOH). The resulting SL-derived phenoxyl radicals are resonance-stabilized and less reactive, effectively slowing the propagation phase of lipid oxidation [47]. This activity parallels the mechanism of natural tocopherols, such as vitamin E, which also localize at lipid interfaces and efficiently trap peroxyl radicals [47]. However, SL offers additional advantages due to its tailored fatty acid chain, which improves its residency within the oil phase and enhances its resistance to volatilization and thermal degradation compared to conventional phenolics.

When compared to butylated hydroxytoluene (BHT), a common synthetic antioxidant, SL exhibits several notable differences. BHT contains a single phenolic hydroxyl group protected by bulky tert-butyl groups, which stabilize the phenoxyl radical but limit its reactivity. While BHT is effective at low temperatures, it can decompose or volatilize under high-heat frying conditions, reducing its protective capacity [44]. In contrast, SL’s multi-phenolic core provides multiple sites for radical quenching, while the linoleate moiety ensures stronger interaction with the lipid matrix, leading to sustained protection even during prolonged heating.

Compared to tocopherols, which are natural, lipid-soluble antioxidants widely present in vegetable oils, SL demonstrates a similar localization behavior at lipid interfaces but with a distinct structural advantage. The presence of multiple phenolic sites in SL allows for multi-point radical scavenging, whereas tocopherols primarily rely on a single chromanol hydroxyl group. Moreover, tocopherols can be regenerated by other reducing agents such as ascorbate, whereas SL is not readily regenerated, making its initial positioning and stability within the oil matrix even more crucial for its antioxidant performance [47].

In summary, the superior efficacy of SL in inhibiting thermal oxidation of sunflower oil can be attributed to its synergistic structural features, which are (i) a polyphenolic core providing strong radical-trapping capacity and (ii) a hydrophobic fatty acid chain enhancing interfacial localization and persistence within the oil phase. This dual action differentiates SL from traditional antioxidants such as BHT and tocopherols, positioning it as a promising natural alternative for improving the oxidative stability of edible oils under industrial processing and frying conditions.

### 3.4. Nutritional Indices for Assessing Fatty Acid Composition

**Figure 9:**
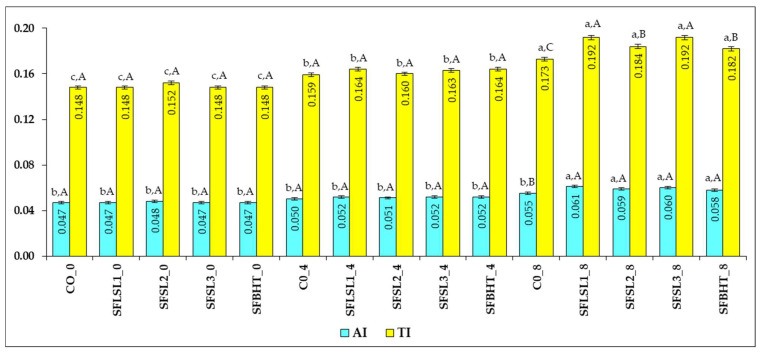
AI (Atherogenicity Index) and TI (Thrombogenicity Index) of oil samples (control—CO; antioxidant-supplemented—SFSL1, SFSL2, SFSL3, SFBHT) in response to heat exposure. Values represent the mean of three independent analyses ± standard deviation (SD). Bars with different letters are statistically different according to one-way ANOVA (*p* < 0.05). Lowercase letters indicate differences among samples with the same antioxidant dose across different thermal treatment durations (0, 4, and 8 h), whereas uppercase letters indicate differences among antioxidant-supplemented samples within the same exposure period.

In order to evaluate the beneficial potential on health, functional indices were calculated, according to the equations presented in Section 2.9. Figure 9 shows the AI (Atherogenic Index) and TI (Thrombogenic Index) values for various analyzed samples (CO, SFSL2, SFSL3, SFLSL1, BHT, C8), at different times (0, 4 and 8).

AI reflects the balance between saturated and unsaturated fatty acids, indicating the potential impact of dietary fats on cardiovascular health [48]. Saturated fatty acids (SFAs) such as C12:0, C14:0, and C16:0—excluding C18:0—are classified as pro-atherogenic, as they promote lipid adhesion to the cells of the circulatory and immune systems [49]. In contrast, unsaturated fatty acids (UFAs) are considered anti-atherogenic, due to their role in preventing plaque buildup and reducing levels of phospholipids, cholesterol, and esterified fatty acids [50]. Consequently, a lower AI value is associated with a reduced risk of elevated total cholesterol and LDL-C levels in human blood plasma [51]. AI provides an early indication of the potential for accelerated atherosclerosis, offering insights into the various inflammatory pathways involved in its development. In parallel, the TI reflects the propensity for blood clot formation, serving as a predictor of thrombotic events and cardiovascular disease risk [52].

AI varies between 0.047 (CO_0, SFSL2_0, BHT_0) and 0.061 (C8); all values are very low, significantly lower than those reported for animal fats (0.3–1.0) and even in the lower range of the typical range for vegetable oils with a healthy profile (0.05–0.20) [52]. This indicates a favorable atherogenic MUFA+PUFA/SFA ratio, with a high cholesterol-lowering potential.

For TI, the values are very low, well below those typical for animal products (0.6–1.0) and partially hydrogenated oils (>0.3), ranging between 0.148 (CO_0, BHT_0) and 0.192 (SFSL2_8, C8). Higher values at time 8 (e.g., SFSL2_8, C8, BHT_8) suggest a slight increase in the proportion of thrombogenic saturated fatty acids (C14:0, C16:0, C18:0) or a decrease in anti-thrombotic PUFAs, especially n-3. AI and TI are positively correlated, samples with higher AI tend to have higher IT. The simultaneous increase in both indices, observed especially at time 8 in some samples, may indicate an unfavorable change in the lipid profile over time, although the absolute values remain in the optimal range.

The low values of AI and TI in all samples suggest a cardioprotective lipid profile—characterized by high content of monounsaturated fatty acids (e.g., oleic acid), moderate content of polyunsaturated fatty acids with an anti-inflammatory effect, and low concentration of saturated fatty acids with an atherogenic and thrombogenic effect.

SFSL (all variants) starts from AI 0.047–0.048 and, even if there are small increases at time 8 (maximum 0.059 at SFSL2_8), these are smaller than the increase observed with BHT and maintain AI in the optimal area (<0.06). These findings suggest that SFSL limits the increase in atherogenic fatty acids better than BHT and maintains values close to CO, indicating an antiatherogenic effect comparable to the control and superior to BHT.

Regarding TI evolution, SFSL has a smaller and more controlled increase in IT compared to BHT, and certain SFSL variants (e.g., SFSL1_4, SFSL3_4) maintain values very close to the control, indicating a partial antithrombogenic effect.

CO (control) and SFSL1/SFSL2/SFSL3 at time 0 present very close values for AI (0.047–0.048) and TI (0.148–0.152), which indicates an almost identical initial lipid profile very favorable for cardiovascular health. BHT and C8 generally have slightly higher AI and TI values, especially at time 8, which suggests a slight deterioration of the fatty acid profile towards a higher content of atherogenic and thrombogenic SFAs.

SFSL, in all variants and times, maintains low values of AI and TI, without large increases towards the end, unlike BHT and C8, where an increasing trend is observed at time 8. The AI remains in the range of 0.047–0.052 for SFSL, which is well below the thresholds considered risky (>0.2).

Compared to BHT_8 (AI = 0.058) and C8 (AI = 0.061), SFSL2_8 (AI = 0.059) and SFSL3_4 (AI = 0.052) have lower or similar values, indicating a better capacity to maintain an optimal atherogenic MUFA+PUFA/SFA ratio. This suggests an obvious antiatherogenic effect, by limiting the increase in fatty acids with atherogenic effect (C14:0, C16:0) over time.

TI for SFSL is maintained between 0.152 and 0.192, which, although slightly higher at time 8 compared to time 0, remains low compared to typical values for lipids with an unfavorable profile (>0.3). The increase in IT at SFSL2_8 (0.192) is similar to C8 (0.192), but SFSL3_4 and SFSL1_4 have reduced values (~0.163–0.164), suggesting that certain SFSL variants better maintain the proportion of anti-thrombotic PUFAs, especially n-3. This behavior suggests a moderate antithrombogenic effect, more pronounced at certain variants and times.

Overall, compared to BHT and C8, SFSL maintains a more stable and protective lipid profile throughout the study, suggesting a positive influence on reducing atherosclerotic and thrombotic risk.

**Figure 10:**
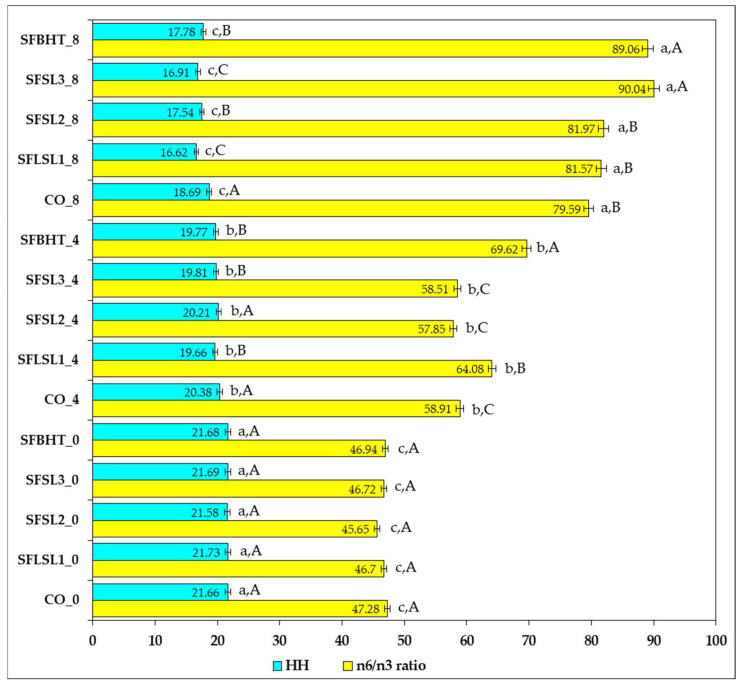
The variation in the n-6/n-3 ratio and HH values of oil samples (control—CO; antioxidant-supplemented—SFSL1, SFSL2, SFSL3, SFBHT) in response to heat exposure. Values represent the mean of three independent analyses ± standard deviation (SD). Bars with different letters are statistically different according to one-way ANOVA (*p* < 0.05). Lowercase letters indicate differences among samples with the same antioxidant dose across different thermal treatment durations (0, 4, and 8 h), whereas uppercase letters indicate differences among antioxidant-supplemented samples within the same exposure period.

Figure 10 shows the variation in the n-6/n-3 ratio and HH values for different samples (CO, SFLSL, SFSL, SFBHT) analyzed at three time points or conditions (0, 4, and 8).

The n-6/n-3 ratio values vary significantly between samples, from 45.65 (SFSL2_0) to 90.04 (SFSL3_8). Moderate values (≈45–65) are recorded in most samples at time point 0 and partially at 4, suggesting a relatively better balance between omega-6 (excessive pro-inflammatory) and omega-3 (anti-inflammatory) fatty acids [53]. High values (>80), such as those observed at time 8 for SFLSL1, SFSL2, SFSL3 and SFBHT, indicate a marked imbalance in favor of omega-6, which according to the literature may be associated with a higher risk of chronic low-grade inflammation and cardiovascular pathology [54]. The optimal ratio recommended by the FAO/WHO (2010) is ≤5:1, and values of 15–20:1 are observed in typical Western diets. Samples with values above 70:1 significantly exceed these limits, indicating an unbalanced proportion of lipid intake [55].

The HH indicator (hypocholesterolemic/hypercholesterolemic) represents the ratio between fatty acids that lower cholesterol (MUFAs and PUFAs) and those that increase it (SFAs with atherogenic effect). In the presented data, HH ranges from ~16.6 (SFLSL1_8) to ~21.8 (SFLSL1_0). Although all values are relatively close, the general trend shows a slight decrease in HH between time 0 and time 8 in most samples, suggesting a relative increase in the proportion of saturated fatty acids over time or under the effect of the experimental treatment.

A strong direct correlation between HH and n-6/n-3 is not observed in this dataset; samples with higher HH do not necessarily have an optimal n-6/n-3 ratio. This emphasizes that the balance between omega-6 and omega-3 and the SFA/MUFA/PUFA ratio are two independent parameters that must be followed simultaneously to assess the nutritional quality of lipids [50].

The ratio of polyunsaturated fatty acids to saturated fatty acids (PUFAs/SFAs) is an essential nutritional indicator for assessing the lipid quality of foods, particularly regarding its impact on cardiovascular health. A high ratio is considered beneficial, as it is associated with a reduction in total cholesterol and LDL-C and with a favorable lipid profile [30].

**Figure 11:**
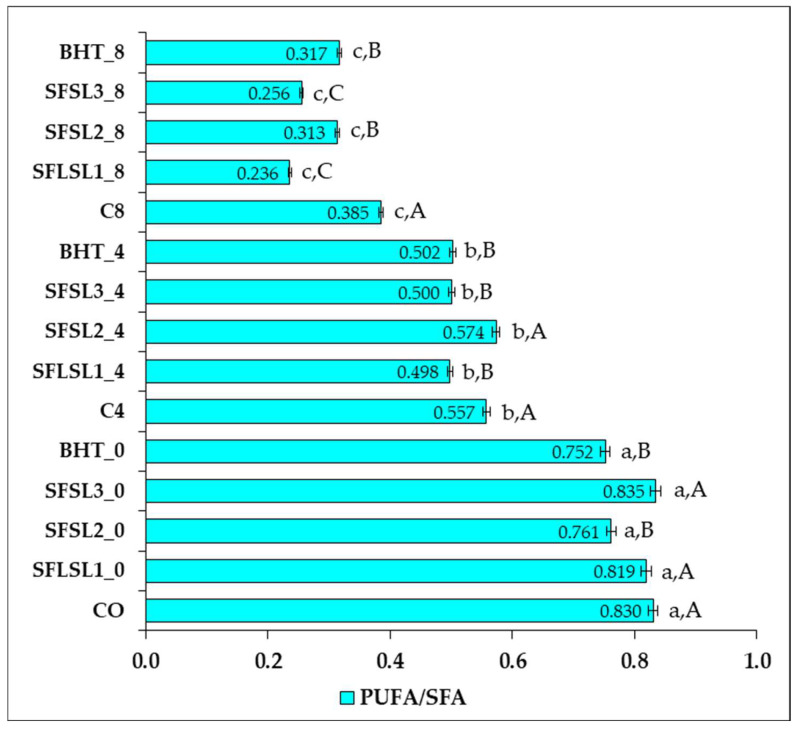
Variation in PUFA/SFA ratios of oil samples (control—CO; antioxidant-supplemented—SFSL1, SFSL2, SFSL3, SFBHT) after 4 and 8 h of heat exposure. Values represent the mean of three independent analyses ± standard deviation (SD). Bars with different letters are statistically different according to one-way ANOVA (*p* < 0.05). Lowercase letters indicate differences among samples with the same antioxidant dose across different thermal treatment durations (0, 4, and 8 h), whereas uppercase letters indicate differences among antioxidant-supplemented samples within the same exposure period.

In the analyzed samples (Figure 11), a progressive decrease in PUFA/SFA values was observed as the duration of thermal treatment (180 °C) increased from 0 to 8 h, confirming the accelerated thermal degradation of polyunsaturated fatty acids. At the zero moment (thermally unprocessed samples), all variants of oil exhibited high values of the PUFA/SFA ratio, ranging from 0.752 to 0.835. The highest value was recorded for the sample SFSL3_0 (0.835), indicating that the addition of SL at a concentration of 600 ppm does not negatively affect the initial lipid profile but maintains the optimal proportion between PUFAs and SFAs. The samples supplemented with BHT (SFBHT_0) and SL at 200–400 ppm also had high values (≥0.75), comparable to the control CO_0 (0.830). After 4 h of thermal treatment, the PUFA/SFA values significantly decreased in all samples; however, important differences were observed between the types of antioxidants. The sample supplemented with 400 ppm SL (SFSL2_4) recorded the best value (0.574), slightly higher than the thermal control CO_4 (0.557). Additionally, the samples SFSL3_4 and SFBHT_4 had similar values (0.500–0.502), while SFSL1_4 (200 ppm) had a slightly lower value (0.498). These results suggest that SL exerts a concentration-dependent protective effect, optimal at 400 ppm, being comparable to or slightly superior to BHT in preventing medium-term PUFA degradation. After 8 h of thermal treatment, a sharp decrease in the PUFA/SFA ratio was observed in all samples, signaling severe oxidation of the polyunsaturated fraction. The control sample CO_8 recorded a value of 0.385, while the other samples varied between 0.236 (SFSL1_8) and 0.317 (SFBHT_8). Although the addition of BHT provided slightly better protection than SL at 200 and 600 ppm, all values were below the threshold of 0.4, indicating limited antioxidant efficiency under prolonged thermal stress. Overall, the analysis of the PUFA/SFA values reveals that all samples suffer from thermal degradation of PUFAs, but antioxidants can slow down this process. SL at 400 ppm proved to be the most effective natural variant in maintaining the PUFA/SFA ratio over time. In contrast, after 8 h of thermal treatment, the protective effect of antioxidants decreases significantly, probably due to the thermal degradation of active compounds. This imbalance may reduce the potential benefits of a profile rich in MUFAs and PUFAs and should be corrected by increasing omega-3 intake (e.g., ALA, EPA, DHA) or reducing omega-6 intake [30].

## 4. Conclusions

In conclusion, silibinin linoleate (SL) demonstrated a dose-dependent protective effect against thermal oxidation in sunflower oil. At 600 ppm, SL showed comparable or superior efficacy to BHT, as reflected by lower PV, p-AV, and TOTOX values, while preserving a more stable fatty acid profile. However, this effect was most evident at the highest concentration tested, and it does not imply superiority across all concentrations. While all tested samples maintained favorable nutritional indices, some minor changes were observed after extended heating, such as a slight increase in atherogenic and thrombogenic indices and a decrease in unsaturated fatty acids, particularly PUFAs.

These findings support the potential of SL as a natural antioxidant for improving the oxidative stability of edible oils. Nevertheless, further studies are required to (i) evaluate the efficacy of lower doses (100–400 ppm), (ii) optimize delivery systems to improve its dispersion and efficiency, and (iii) extend investigations to real food matrices to confirm its practical applicability.

In addition, future research should incorporate advanced analytical approaches, such as GC-MS or HPLC-MS/MS, to quantify oxidation-specific products (e.g., 4-HNE, MDA, hydroxy fatty acids) and integrate these with FTIR and chromatographic data. This will enable the establishment of quantitative relationships between PUFA degradation, oxidation indices, and product distribution, thereby providing a more comprehensive understanding of SL’s antioxidant mechanism and its potential role in food systems.

## Figures and Tables

**Table 1:** The experimental samples and the treatment applied.

No.	Abbreviation	Composition	Heating Treatment
1	CO_0	Sunflower oil (SF)	No heating
2	SFSL1_0	Sunflower oil (SF) + 200 ppm silibinin linoleate (SL)	No heating
3	SFSL2_0	Sunflower oil (SF) + 400 ppm silibinin linoleate (SL)	No heating
4	SFSL3_0	Sunflower oil (SF) + 600 ppm silibinin linoleate (SL)	No heating
5	SFBHT_0	Sunflower oil (SF) + 200 ppm BHT	No heating
6	CO_4	Sunflower oil (SF)	4 h at 180 °C
7	SFSL1_4	Sunflower oil (SF) + 200 ppm silibinin linoleate (SL)	4 h at 180 °C
8	SFSL2_4	Sunflower oil (SF) + 400 ppm silibinin linoleate (SL)	4 h at 180 °C
9	SFSL3_4	Sunflower oil (SF) + 600 ppm silibinin linoleate (SL)	4 h at 180 °C
10	SFBHT_4	Sunflower oil (SF) + 200 ppm BHT	4 h at 180 °C
11	CO_8	Sunflower oil (SF)	8 h at 180 °C
12	SFSL1_8	Sunflower oil (SF) + 200 ppm silibinin linoleate (SL)	8 h at 180 °C
13	SFSL2_8	Sunflower oil (SF) + 400 ppm silibinin linoleate (SL)	8 h at 180 °C
14	SFSL3_8	Sunflower oil (SF) + 600 ppm silibinin linoleate (SL)	8 h at 180 °C
15	SFBHT_8	Sunflower oil (SF) + 200 ppm BHT	8 h at 180 °C

**Table 2:** Changes in peroxide value (PV) and para-anisidine value (p-AV) of sunflower oil samples following supplementation with SL and BHT, before and after 4 and 8 h of heat exposure, compared to the control (C).

Sample	PV (meq O_2_//kg oil)	p-AV	IO (%)	TOTOX
		No heating		
CO_0	1.81 ± 0.06 ^a^	2.97 ± 0.05 ^a^	-	6.59 ± 0.17 ^a^
SFSL1_0	1.79 ± 0.08 ^a^	2.96 ± 0.08 ^a^	-	6.54 ± 0.11 ^a^
SFSL2_0	1.77 ± 0.04 ^a^	2.94 ± 0.07 ^a^	-	6.48 ± 0.16 ^a^
SFSL3_0	1.74 ± 0.05 ^a^	2.90 ± 0.06 ^a^	-	6.38 ± 0.19 ^a^
SFBHT_0	1.76 ± 0.04 ^a^	2.93 ± 0.04 ^a^	-	6.45 ± 0.15 ^a^
		4 h at 180 °C		
CO_4	21.78 ± 0.31 ^a^	30.47 ± 0.47 ^a^	-	74.03 ± 0.54 ^a^
SFSL1_4	18.18 ± 0.29 ^b^	26.34 ± 0.41 ^b^	17.91 ± 0.16 ^a^	62.70 ± 0.61 ^b^
SFSL2_4	14.35 ± 0.24 ^c^	21.24 ± 0.35 ^c^	36.99 ± 0.29 ^b^	49.95 ± 0.51 ^c^
SFSL3_4	9.72 ± 0.19 ^d^	18.09 ± 0.28 ^d^	60.03 ± 0.34 ^c^	37.53 ± 0.45 ^d^
SFBHT_4	13.73 ± 0.19 ^e^	19.56 ± 0.39 ^e^	40.10 ± 0.32 ^d^	47.01 ± 0.42 ^e^
		8 h at 180 °C		
CO_8	30.45 ± 0.56 ^a^	41.07 ± 0.43 ^a^	-	101.98 ± 0.65 ^a^
SFSL1_8	24.55 ± 0.37 ^b^	34.16 ± 0.48 ^b^	20.52 ± 0.19 ^a^	83.27 ± 0.45 ^b^
SFSL2_8	18.25 ± 0.33 ^c^	27.03 ± 0.41 ^c^	42.48 ± 0.21 ^b^	63.24 ± 0.52 ^c^
SFSL3_8	14.22 ± 0.31 ^d^	22.85 ± 0.34 ^d^	56.41 ± 0.31 ^c^	51.30 ± 0.39 ^d^
SFBHT_8	17.09 ± 0.35 ^e^	25.58 ± 0.39 ^e^	46.48 ± 0.26 ^d^	59.77 ± 0.048 ^e^

Values are presented as means of three replicates ± standard deviation. Data in the same column, for each group of treatment (initial, after 4 and 8 h of heating at 180 °C) with different superscript letters are significantly different (*p* < 0.05), while those sharing the same letter are not significantly different (*p* > 0.05). For sample information, see Table 1.

**Table 3:** Fatty acid composition of oil samples with and without antioxidants before and after heating (180 °C; 4 h and 8 h).

Fatty Acid		CO_0	SFLSL1_0	SFSL2 _0	SFSL3 _0	SFBHT_0	CO_4	SFLSL1_4	SFSL2 _4	SFSL3 _4	SFBHT_4	CO_8	SFLSL1_8	SFSL2 _8	SFSL3 _8	SFBHT_8
C14.0	Myristic acid	0.045 ± 0.0034 ^a^	0.043 ± 0.0056 ^a^	0.042 ± 0.0008 ^a^	0.045 ± 0.0034 ^a^	0.046 ± 0.0019 ^a^	0.044 ± 0.0027 ^b^	0.044 ± 0.002 ^b^	0.042 ± 0.0033 ^b^	0.042 ± 0.0018 ^b^	0.049 ± 0.0001 ^a^	0.043 ± 0.005 ^b^	0.043 ± 0.0034 ^b^	0.045 ± 0.0016 ^b^	0.031 ± 0.0002 ^c^	0.063 ± 0.0025 ^a^
C16:0	Palmitic acid	3.982 ± 0.0442 ^a^	3.839 ± 0.0354 ^b^	3.750 ± 0.1187 ^b^	4.029 ± 0.0225 ^a^	4.031 ± 0.1475 ^a^	3.958 ± 0.115 ^b^	3.85 ± 0.012 ^b^	3.696 ± 0.081 ^b^	3.76 ± 0.068 ^b^	4.3083 ± 0.0958 ^a^	4.476 ± 0.075 ^a^	3.549 ± 0.0527 ^d^	3.8417 ± 0.0197 ^c^	4.6177 ± 0.1184 ^a^	4.304 ± 0.033 ^b^
C16:1	Palmitoleic acid	0.115 ± 0.0032 ^a^	0.111 ± 0.0051 ^b^	0.010 ± 0.0008 ^c^	0.116 ± 0.0067 ^a^	0.116 ± 0.005 ^a^	0.109 ± 0.0022 ^a^	0.104 ± 0.0072 ^b^	0.037 ± 0.0026 ^c^	0.102 ± 0.0021 ^b^	0.12 ± 0.0059 ^a^	0.116 ± 0.0042 ^a^	0.087 ± 0.0033 ^b^	0.097 ± 0.011 ^b^	0.048 ± 0.002 ^c^	0.038 ± 0.0033 ^c^
C17.0	Margaric acid	0.032 ± 0.0024 ^a^	0.029 ± 0.0026 ^a^	0.029 ± 0.003 ^a^	0.031 ± 0.0005 ^a^	0.032 ± 0.0016 ^a^	0.045 ± 0.003 ^a^	0.036 ± 0.0033 ^b^	0.032 ± 0.0019 ^b^	0.039 ± 0.0008 ^a^	0.043 ± 0.0048 ^a^	0.046 ± 0.0034 ^a^	0.029 ± 0.0008 ^d^	0.04 ± 0.0028 ^b^	0.046 ± 0.0052 ^a^	0.05 ± 0.0008 ^a^
C17:1	Heptadecanoic acid	0.052 ± 0.0035 ^a^	0.047 ± 0.0019 ^a^	0.046 ± 0.0034 ^a^	0.049 ± 0.0039 ^a^	0.050 ± 0.0053 ^a^	0.049 ± 0.0031 ^a^	0.011 ± 0.0002 ^c^	0.048 ± 0.0024 ^a^	0.043 ± 0.0038 ^b^	0.051 ± 0.0019 ^a^	0.036 ± 0.0033 ^b^	0.036 ± 0.0023 ^b^	0.042 ± 0.0024 ^b^	0.053 ± 0.002 ^a^	0.048 ± 0.0028 ^a^
C18:0	Stearic acid	2.540 ± 0.0861 ^a^	2.454 ± 0.0445 ^a^	2.522 ± 0.0675 ^a^	2.5673 ± 0.067 ^a^	2.557 ± 0.0352 ^a^	2.573 ± 0.066 ^a^	2.456 ± 0.15 ^b^	2.355 ± 0.1413 ^b^	2.416 ± 0.0555 ^b^	2.781 ± 0.1168 ^a^	2.896 ± 0.0508 ^b^	2.277 ± 0.0478 ^d^	2.452 ± 0.0298 ^c^	2.971 ± 0.038 ^a^	2.745 ± 0.0157 ^b^
C18:1	Oleic acid	81.084 ± 0.8503 ^a^	78.388 ± 0.3359 ^b^	76.033 ± 0.8964 ^c^	82.119 ± 0.0278 ^a^	82.152 ± 0.7496 ^a^	76.956 ± 0.5834 ^b^	72.657 ± 0.145 ^c^	71.258 ± 0.5529 ^d^	71.289 ± 0.3981 ^d^	81.684 ± 0.7542 ^a^	80.924 ± 0.716 ^a^	57.815 ± 0.5992 ^d^	65.673 ± 0.7652 ^c^	76.165 ± 0.7667 ^b^	74.743 ± 0.9816 ^b^
C18:2	Linoleic acid	6.004 ± 0.067 ^a^	5.838 ± 0.207 ^a^	5.661 ± 0.1162 ^a^	6.1203 ± 0.315 ^a^	6.102 ± 0.0036^a^	4.536 ± 0.147 ^a^	3.845 ± 0.0789 ^c^	4.223 ± 0.115 ^b^	3.979 ± 0.1929 ^c^	4.386 ± 0.1045 ^a^	3.502 ± 0.143 ^a^	1.876 ± 0.0871 ^d^	2.459 ± 0.0589 ^c^	2.431 ± 0.0346 ^c^	2.850 ± 0.0496 ^b^
C18:3α	α-Linolenic acid (ALA)	0.127 ± 0.0036 ^a^	0.125 ± 0.008 ^a^	0.124 ± 0.0036 ^a^	0.131 ± 0.0042 ^a^	0.130 ± 0.0011 ^a^	0.077 ± 0.0134 ^a^	0.06 ± 0.0049 ^a^	0.073 ± 0.0097 ^a^	0.068 ± 0.0059 ^a^	0.063 ± 0.0054 ^a^	0.044 ± 0.0047 ^a^	0.023 ± 0.0005 ^c^	0.03 ± 0.0006 ^b^	0.027 ± 0.001 ^b^	0.032 ± 0.0016 ^b^
C20:0	Arichidic acid	0.260 ± 0.0077 ^a^	0.250 ± 0.0049 ^a^	0.247 ± 0.0198 ^a^	0.260 ± 0.0089 ^a^	0.263 ± 0.0102 ^a^	0.259 ± 0.0064 ^a^	0.251 ± 0.0074 ^b^	0.240 ± 0.0188 ^b^	0.254 ± 0.0108 ^b^	0.281 ± 0.0199 ^a^	0.294 ± 0.0045 ^a^	0.227 ± 0.0062 ^c^	0.245 ± 0.0067 ^b^	0.298 ± 0.0192 ^a^	0.274 ± 0.0098 ^b^
C20:1	Eicosenoic acid	0.288 ± 0.0152 ^a^	0.282 ± 0.0154 ^a^	0.27 ± 0.013 ^b^	0.291 ± 0.0087 ^a^	0.293 ± 0.0076 ^a^	0.282 ± 0.0185 ^a^	0.254 ± 0.0132 ^b^	0.272 ± 0.0043 ^b^	0.223 ± 0.0078 ^b^	0.296 ± 0.0277 ^a^	0.430 ± 0.0155 ^a^	0.219 ± 0.0044 ^d^	0.249 ± 0.0092 ^c^	0.296 ± 0.0052 ^b^	0.271 ± 0.0059 ^c^
C22:0	Behenic acid	ND	0.021 ± 0.0024 ^b^	0.823 ± 0.0773 ^a^	*ND	0.865 ± 0.0541 ^a^	0.857 ± 0.1177 ^c^	0.853 ± 0.0247 ^b^	0.816 ± 0.0364 ^b^	0.854 ± 0.0184 ^b^	0.927 ± 0.0391 ^a^	0.9771 ± 0.0727 ^a^	0.771 ± 0.0334 ^b^	0.840 ± 0.0402 ^b^	1.043 ± 0.0301 ^a^	0.954 ± 0.0343 ^a^
C22:1	Erucic acid	0.019 ± 0.0017 ^c^	0.017 ± 0.0025 ^c^	0.037 ± 0.0031 ^a^	0.024 ± 0.0001 ^b^	0.020 ± 0.0014 ^b^	0.041 ± 0.0004 ^c^	0.121 ± 0.0066 ^a^	0.084 ± 0.0026 ^b^	0.059 ± 0.003 ^d^	0.066 ± 0.003 ^c^	0.155 ± 0.0043 ^a^	0.113 ± 0.0034 ^c^	0.129 ± 0.006 ^b^	0.089 ± 0.0063 ^d^	0.132 ± 0.0057 ^b^
C24:0	Lignoceric acid	0.525 ± 0.0385 ^b^	0.641 ± 0.029 ^a^	0.184 ± 0.016 ^c^	0.556 ± 0.039 ^b^	0.488 ± 0.0422 ^b^	0.54 ± 0.0131 ^b^	0.348 ± 0.0239 ^d^	0.307 ± 0.014 ^d^	0.726 ± 0.0249 ^a^	0.469 ± 0.0315 ^c^	0.475 ± 0.0199 ^d^	1.163 ± 0.0337 ^a^	0.485 ± 0.0255 ^d^	0.613 ± 0.0349 ^c^	0.712 ± 0.0165 ^b^
C24:1	Nervonic acid	0.117 ± 0.0032 ^a^	*ND	*ND	*ND	0.098 ± 0.0159 ^a^	0.233 ± 0.0166 ^a^	*ND	*ND	0.168 ± 0.0083 ^d^	*ND	0.071 ± 0.0049 ^b^	0.829 ± 0.0508 ^a^	*ND	0.252 ± 0.0154 ^d^	*ND

*ND = not detected. Values are expressed as mean ± standard deviation (n = 3). The statistical analysis performed include different superscript letters (e.g., a, b, c, d) within the same row, and the time point indicates significant differences between samples according to one-way ANOVA followed by Tukey’s HSD post hoc test (*p* < 0.05).

## Data Availability

The original data presented in the study are openly available at the University of Life Sciences “King Mihai I” from Timișoara.
